# Transdiagnostic and clinical predictors of longitudinal misophonia symptoms

**DOI:** 10.3389/fcogn.2026.1945899

**Published:** 2026-10-05

**Authors:** Jeffrey M. Pavlacic, Averi N. Gaines, Sara M. Witcraft, Allie Freshley, Erin M. Buchanan, Bernadette Cortese, Christopher Sege, Alyssa A. Rheingold, Thomas Uhde, Laura J. Dixon

**Affiliations:** 1 Department of Psychiatry and Behavioral Sciences, Medical University of South Carolina, Charleston, SC, United States; 2 Department of Psychology, University of Mississippi, University, MS, United States; 3 Analytics, Harrisburg University of Science and Technology, Harrisburg, PA, United States

**Keywords:** anxiety sensitivity, distress tolerance, ecological momentary assessment, emotion dysregulation, misophonia

## Abstract

**Introduction:**

Anxiety sensitivity (AS) and distress tolerance (DT) are emotional-vulnerability factors driving emotion dysregulation. AS and DT may amplify misophonia. Research examining transdiagnostic factors predicting misophonia is largely cross-sectional. We examined relations between AS, DT, and longitudinal misophonia symptoms, adjusting for clinical variables.

**Methods:**

Fifty-eight individuals recruited from soQuiet endorsing clinical misophonia symptoms completed baseline and daily surveys 3x daily for 30 days.

**Results:**

Bivariate analyses suggested baseline AS was associated with elevated longitudinal misophonia symptoms during and not during triggers, while baseline DT was associated with higher misophonia symptoms during triggers. Baseline AS, momentary emotion dysregulation, and baseline misophonia were predictors of longitudinal misophonia during triggers, adjusting for momentary emotion dysregulation, baseline misophonia, and baseline obsessions and compulsions. An alpha correction suggested an attenuated AS effect. Null effects for DT may be explained by analyses supporting emotion dysregulation as a mediator of the relation between DT and misophonia. Effects of obsessive-compulsive symptoms were null.

**Discussion:**

Results inform longitudinal relations between transdiagnostic factors and misophonia. A predominant limitation is sample homogeneity. Misophonia symptom fluctuations appear contextualized, likely impacted by environmental and emotional-vulnerability factors. Results inform ongoing advancements to misophonia assessment and, pending further investigation, clinical interventions targeting malleable transdiagnostic factors.

## 1 Introduction

Misophonia is characterized by decreased tolerance to specific sounds and related stimuli (Swedo et al., 2022). United States epidemiological studies report a misophonia prevalence rate of ~5% (Dixon et al., 2024). Central to misophonia are “triggers,” specific sounds (e.g., chewing) or stimuli related to aversive sounds (Swedo et al., 2022). Triggers are repetitive or rhythmic and contribute to affective, behavioral, and cognitive responses (Rosenthal et al., 2022). Etiological misophonia correlates include neurological factors (Neacsiu et al., 2022), psychological mechanisms (Cowan et al., 2022), and social variables (see Berger et al., 2024). Existing misophonia research has examined cross-sectional correlates and sought to operationalize misophonia via expert consensus (Swedo et al., 2022). Less research has used longitudinal methods to identify longitudinal misophonia predictors to understand symptom expression.

Patterns of moment-to-moment misophonia reactivity and recovery vary individually and contribute to fluctuations in negative affect (e.g., anger; Shan et al., 2026). Momentary misophonia symptom variability is supported in longitudinal investigations using ecological momentary assessment (EMA; Pavlacic et al., 2026). EMA misophonia studies suggest (1) misophonia symptom expression varies individually; and (2) momentary emotional states and psychological factors covary with momentary misophonia symptoms. Examining the relative role of transdiagnostic factors in predicting misophonia symptoms could inform an understanding of malleable misophonia symptom amplifiers and attenuators.

Transdiagnostic correlates, such as distress tolerance (ability to tolerate and manage distress [DT]; Zvolensky et al., 2010) and anxiety sensitivity (fear of anxiety-related sensations [AS]; Olatunji and Wolitzky-Taylor, 2009; Reiss et al., 1986), are well-established emotional-vulnerability factors facilitating anticipatory distress (Schadegg et al., 2021) and difficulty responding to emotion-inducing cues (Kashdan et al., 2008; Leyro et al., 2010; Mattingley et al., 2022; Naragon-Gainey, 2010; Olatunji and Wolitzky-Taylor, 2009). AS and DT are malleable and clinical targets (Reese et al., 2019; Schmidt et al., 2014). AS and DT synergistically influence the way someone processes, reacts to, and manages emotions in real-time (e.g., Gross et al., 2026), potentially exacerbating misophonia symptoms. DT, for example, may contribute to reinforced avoidance behaviors (Jeffries et al., 2016; San Martín et al., 2020; Zvolensky et al., 2010).

AS may separately facilitate hypervigilance of physiological trigger sequelae (Olatunji and Wolitzky-Taylor, 2009), leading to behavioral escape or avoidance. AS is a risk factor for anxiety (Schmidt et al., 1997). Both AS and DT are independently associated with greater misophonia symptoms; increasing DT may improve misophonia symptoms (Cepeda et al., 2026). Despite models linking DT, AS, and misophonia symptoms, limited research has examined whether DT and AS predict misophonia longitudinally in adults.

### 1.1 Present study

To address longitudinal misophonia assessment gaps, three aims were investigated through secondary analyses. The primary outcome paper/study can be found at (Pavlacic et al., 2026). We examined baseline clinical correlates of longitudinal misophonia symptoms (trait-level misophonia; obsessive-compulsive symptoms) during and not during triggers throughout an EMA study (*Aim 1*). We tested bivariate relations between transdiagnostic risk factors (AS and DT) and longitudinal misophonia symptoms experienced during and not during triggers (*Aim 2*).

To examine the role of AS and DT in predicting longitudinal misophonia (*Aim 3*), we tested AS and DT as predictors of longitudinal misophonia symptoms in a comprehensive model relative to bivariate analyses. *Aim 3* multilevel models adjusted for trait-level misophonia, obsessive-compulsive symptoms (Cusack et al., 2018), and within- and between-person emotion dysregulation (Pavlacic et al., 2026; Weiss et al., 2025).

Exploratory mediation analyses were tested to elucidate indirect effects (or lack thereof) of emotion dysregulation in relations between AS/DT and misophonia. Analyses were conducted separately during and not during triggers, given heightened anger and irritability during trigger sounds (Shan et al., 2026) and unique misophonia subtypes (anticipatory vs. reactive; Woolley et al., 2025).

## 2 Materials and methods

### 2.1 Participants and procedure

The study protocol received Institutional Review Board approval. Analyses are secondary analyses from a larger study (Pavlacic et al., 2026). To summarize, participants from the soQuiet (i.e., non-profit organization focused on misophonia support, awareness, and resources) research pool were invited to participate in a screener to assess eligibility. Exclusions were reporting a diagnosis of autism spectrum disorder or psychotic disorder. Participants were adults 18+ screening positive for misophonia via the Duke-Vanderbilt Misophonia Screening Questionnaire (DVMSQ; Williams et al., 2022). Following eligibility determination, participants were enrolled in Inclivio to complete the daily portion, lasting 30 days. Participants completed interval-contingent and event-contingent (based on trigger experience) assessments of misophonia symptoms 3x daily for 30 days. Participants were instructed to record event-contingent responses using prior survey links sent through Inclivio.

### 2.2 Baseline measures

#### 2.2.1 Demographics

Participants provided demographics (e.g., age, education).

#### 2.2.2 Anxiety Sensitivity Index

The Anxiety Sensitivity Index (ASI; Reiss et al., 1986) is a 16-item self-report measure administered on a 5-point Likert-type scale measuring fear of physical anxiety sensations and associated beliefs. Higher summed scores suggest more elevated AS. The ASI is psychometrically supported (Reiss et al., 1986) and was administered at baseline. Internal consistency alpha was good at 0.88.

#### 2.2.3 Distress Tolerance Scale

The Distress Tolerance Scale (DTS; Simons and Gaher, 2005) is a 15-item self-report measure administered on a 5-point Likert-type scale measuring perceived ability to tolerate distress. Higher total scores suggest greater distress *intolerance*. The DTS is psychometrically supported (Simons and Gaher, 2005) and demonstrated excellent baseline internal consistency at 0.93.

#### 2.2.4 Obsessive-Compulsive Inventory-Revised

The Obsessive-Compulsive Inventory-Revised (OCI-R; Abramowitz and Deacon, 2006) is an 18-item self-report administered on a 5-point Likert-type scale. Higher scores suggest greater OC symptoms. The OCI-R is psychometrically supported (Abramowitz and Deacon, 2006) and demonstrated good baseline internal consistency (0.89).

#### 2.2.5 Amsterdam Misophonia Scale

The Amsterdam Misophonia Scale (A-MISO-S; Schröder et al., 2013) is a 6-item self-report measure designed as an adaptation of the Yale-Brown Obsessive Compulsive Scale (Goodman et al., 1989). Greater summed scores suggest higher trait misophonia symptoms. The A-MISO-S has excellent psychometrics (Schröder et al., 2013), and baseline internal consistency alpha was acceptable at 0.70.

### 2.3 Daily measures

#### 2.3.1 Triggers

Trigger presence was assessed with the following adapted prompt (Williams et al., 2022), leading to a set of questions assessing misophonia experience during triggers or questions regarding general misophonia symptoms despite currently not experiencing triggers: “*Are there specific sounds that you are extremely bothered by RIGHT NOW, even if they are not loud? Examples include: chewing, slurping, crunching, throat clearing, finger tapping, foot shuffling, keyboard tapping, rustling, nasal sounds, pen clicking, appliance humming, clock ticking, and animal sounds. These sounds should cause significant emotional distress (e.g., extreme irritation, anger, disgust, rage, anxiety, or panic). Do NOT count sounds that bother you only because you find them too loud or physically painful*.” If “Yes” was endorsed, participants completed the set of questions tailored to triggers. If “No,” an adapted question set was administered asking about general misophonia symptoms.

#### 2.3.2 Momentary misophonia

To measure in-the-moment misophonia symptoms, an adapted version of the DVMSQ (Williams et al., 2022) was created from the original measure. The phrasing “Right now” was added prior to items. Participants were encouraged to respond about experiences either (1) during triggers; or (2) even though they were not currently being triggered. Branching logic was used for specific question sets following triggers. Data were analyzed separately for trigger presence and absence (Shan et al., 2026; Woolley et al., 2025). Summed scores were calculated adhering to Williams et al. (2022). Multilevel composite reliability suggested the adapted measure symptom items exhibited strong reliability at both the within (ω = 0.87) and between (ω = 0.92) levels during triggers, which was also the case not during triggers at within (ω = 0.90) and between (ω = 0.94) levels.

#### 2.3.3 Momentary emotion dysregulation

To measure momentary emotion dysregulation, we used the Momentary Difficulties in Emotion Regulation Scale (mDERS; Weiss et al., 2025). The mDERS is a 6-item measure assessing various domains of emotion dysregulation abilities on a 4-point Likert-type scale. Domains assessed include goals, impulse control difficulties, strategies, awareness, clarity, and non-acceptance. Respondents responded based on experiences since the prior survey. The mDERS has excellent psychometrics (Weiss et al., 2025). Higher scores reflect greater emotion dysregulation. The mDERS was used as a control in MLMs (at both the between- and within-person levels), given relations between emotion dysregulation and misophonia (Pavlacic et al., 2026). Multilevel composite reliability suggested that the mDERS exhibited suboptimal reliability at both the within *(*ω = 0.60) and excellent reliability at the between (ω = 0.88) level.

### 2.4 Data-analytic plan

#### 2.4.1 Aims 1 and 2: between-person misophonia symptom scores

No analyses were pre-registered. All analyses were secondary, calculated as part of a larger study (Pavlacic et al., 2026). Analyses were conducted in *JASP* and *R*. To calculate overall levels of misophonia symptoms throughout the course of the study as an outcome predicted by baseline-factors, each participant's daily scores were averaged to create one overall score (i.e., between-person scores). We examined the correlation of participants' baseline clinical and transdiagnostic characteristics and misophonia symptom severity.

#### 2.4.2 Aim 3: multilevel models

Multilevel models (MLMs) were calculated to examine whether baseline AS, DT, predicted misophonia during triggers and when triggers were absent, controlling for momentary emotion dysregulation at the between- and within-person levels, as well as baseline misophonia severity and baseline obsessive-compulsive symptoms. Within-person emotion dysregulation scores were centered based on an individual's unique mean throughout the longitudinal study component. Between-person scores reflect average emotion dysregulation across momentary surveys.

A random intercept was a better fit relative to a fixed intercept model (*p* < 0.001). The random intercept model was used for the MLM when entering predictors. The *p*-value calculated for the random intercept is based on a Likelihood Ratio. We also considered Akaike Information Criterion and Bayesian Information Criterion (lower values = better fit). Total variance for the trigger model was 209.07, and 144.68 for the no trigger model. Completion of at least one momentary survey was required for MLM inclusion. Missing data were not replaced for longitudinal data, as data were generally missing > 5% when missing for certain measures (i.e., mDERS), leading to different observations across MLMs. The model was:

*Predicted Momentary Misophonia DVMSQ* ~ *Baseline Misphonia (A-MISO-S)* + *Obessessive Compulsive Behaviors (OCI-R)* + *Emotion Dysregulation (mDERS) Within* + *Emotion Dysregulation (mDERS) Between* + *Anxiety Sensitivity (ASI)* + *Distress Intolerance (DTS)* + *(*~*1|Participant), with maximum likelihood estimation and missing data omission*.

The same model was calculated for misophonia during triggers.

#### 2.4.3 Exploratory mediation analyses

Given differential effects for AS, DT, and misophonia in MLMs, we explored momentary emotion dysregulation (between-person scores) as a mediator of the relation between AS/DT and misophonia symptoms both during and not during triggers using a parallel mediation (i.e., mediator on both predictor paths).

## 3 Results

### 3.1 Demographics and survey completion

Sixty participants finished the baseline battery. Most were White (86.7%), non-Hispanic (91.7%), female (88.3%), heterosexual/straight (80.0%), and either single (36.7%) or married (46.7%). Participants resided in either suburban (45.0%) or urban (38.3%) environments. Participants predominantly reported either their income was > $99,999 (50%) or <$10,000 to $75,000-$99,999 (38.3%). Participants completed 2,110 surveys where triggers were not present and 816 with triggers present. Adherence data and descriptive statistics data are described in detail at (Pavlacic et al., 2026). Participants completed on average 50.91 of 90 surveys (*SD* = 31.96, median = 59). Participants reported an average of 14.07 triggers (*SD* = 14.67, range 0–76, median = 9.5).

### 3.2 Aim 1 and 2: correlations with longitudinal misophonia symptoms

Higher levels of baseline misophonia were correlated with greater longitudinal misophonia symptoms during (*r* = 0.553, *p* < 0.001) and not during (*r* = 0.481, *p* < 0.001) triggers. Higher levels of AS were associated with higher misophonia symptoms during (*r* = 0.451, *p* < 0.001) and not during triggers (*r* = 0.314, *p* = 0.018). Higher levels of distress intolerance were correlated with higher misophonia symptom during triggers (*r* = 0.367, *p* = 0.007) but not in the absence of triggers (*r* = 0.245, *p* = 0.069). OC symptoms were positively associated with misophonia symptoms during triggers (*r* = 0.320, *p* = 0.014) and when triggers were absent (*r* = 0.280, *p* = 0.037).

### 3.3 Aim 3: multilevel models

#### 3.3.1 No trigger model

Table 1 provides estimates for omnibus MLMs. In the first model only including data where triggers were not reported, AS and DT were entered simultaneously, controlling for within- and between-person emotion dysregulation, baseline misophonia, and baseline obsessive-compulsive symptoms. At the within-person level, greater momentary emotion dysregulation was associated with higher misophonia symptoms when triggers were absent [*t*(1974) = 10.39, *p* < 0.001, *b* = 0.67, β = 0.12, 95% confidence interval [CI]: 0.10–0.14]. Marginal *R*^2^ was 0.37.

**Table 1:** Estimates for MLMs during and not during triggers for the omnibus model.

No trigger model	SE	β	95% CI	*t*	*p*
Misophonia	0.35	0.34	0.14 to 0.53	3.47	0.001^*^
Obsessive/compulsive	0.13	−0.09	−0.35 to 0.17	−0.73	0.470
Centered regulation	0.06	0.12	0.10 to 0.14	10.39	<0.001^*^
Between regulation	0.30	0.45	0.25 to 0.65	4.84	<0.001^*^
Anxiety sensitivity	0.15	0.15	−0.12 to 0.41	1.11	0.270
Distress intolerance	0.12	−0.09	−0.35 to 0.17	−0.69	0.495
**Trigger model**	**SE**	**β**	**95% CI**	***t***	***p***
Misophonia	0.39	0.30	0.13 to 0.46	3.64	<0.001^*^
Obsessive/compulsive	0.14	−0.13	−0.34 to 0.09	−1.16	0.254
Centered regulation	0.13	0.12	0.09 to 0.15	8.01	<0.001^*^
Between regulation	0.34	0.41	0.26 to 0.57	5.42	<0.001^*^
Anxiety sensitivity	0.16	0.22	0.00 to 0.45	1.97	0.054
Distress intolerance	0.13	−0.04	−0.24 to 0.16	−0.41	0.683

^*^Significant at *p* < 0.05. SE, standard error; β, standardized estimate; CI, confidence interval.

At the between-person level, individuals with greater baseline misophonia severity [*t*(50) = 3.47, *p* = 0.001, *b* = 1.20, β = 0.34, 95% CI 0.14–0.53] and greater emotion dysregulation [*t*(50) = 4.84, *p* < 0.001, *b* = 1.35, β = 0.45, 95% CI 0.25–0.65] reported significantly higher misophonia symptoms when triggers were absent. Baseline AS (β = 0.15, 95% CI: −0.12 to 0.41, *p* = 0.270) and DT (β = −0.09, 95% CI: −0.35-0.17, *p* = 0.495) were not significant predictors of misophonia symptoms. The pattern of results was similar when examining AS (β = 0.10, 95% CI: −0.13 to 0.33, *p* = 0.380) and DT in separate models (β = −0.02, 95% CI: −0.24 to 0.21, *p* = 0.884), which was calculated given the strong correlation between AS and DT (*r* = 0.66).

#### 3.3.2 Triggers

Next, we replicated the same models detailed above when triggers were present. In the first model, AS and DT were entered simultaneously, controlling for within- and between-person emotion dysregulation, baseline misophonia, and baseline obsessive-compulsive symptoms. At the within-person level, greater momentary emotion dysregulation was associated with higher misophonia symptoms when triggers were present [*t*(720) = 8.01, *p* < 0.001, *b* = 1.06, β = 0.12, 95% CI 0.09–0.15]. Marginal *R*^2^ was 0.51.

At the between-person level, individuals with greater baseline misophonia severity [*t*(47) = 3.64, *p* < 0.001, *b* = 1.42, β = 0.30, 95% CI 0.13–0.46] and greater emotion dysregulation [*t*(47) = 5.42, *p* < 0.001, *b* = 1.83, β = 0.41, 95% CI 0.26–0.57] reported higher misophonia symptoms. Baseline AS (*p* = 0.054, β = 0.22, 95% CI 0.00–0.45) and DT (*p* = 0.683, β = −0.04, 95% CI −0.24 to 0.16) were not significant predictors of misophonia symptoms. When examining AS and DT in separate models, calculated given shared variance as evidenced by the correlation between AS and DT, DT was not a significant predictor (*p* = 0.481, β = 0.06, 95% CI −0.11 to 0.24), whereas AS was [*t*(48) = 2.06, *p* = 0.044, *b* = 0.28, β = 0.20, 95% CI 0.01–0.39, model marginal *R*^2^ = 0.51]. When DT was not accounted for, individuals with greater trait-level AS tended to report greater misophonia symptoms during triggers. When employing a Benjamini-Hochberg (BH) correction, the adjusted *p*-value for AS was 0.055.

#### 3.3.3 Exploratory mediation analyses

Given differential results for DT and AS, and potential shared variance given the correlation (0.66), we tested emotion dysregulation as a mediator between DT/AS and misophonia during and not during triggers using parallel mediation. During triggers for DT, the indirect effect of momentary emotion dysregulation was significant (*p* < 0.001, *b* = 0.31, *SE* = 0.02, 95% CI: 0.28–0.32). Regarding AS effects, emotion dysregulation was not a significant mediator during triggers (*p* = 0.199, *b* = 0.02, *SE* = 0.02, 95% CI: −0.01 to 0.05). Mediation results exhibited a similar pattern outside triggers for DT and AS. The indirect effect was significant for momentary emotion regulation for DT (*p* < 0.001, *b* = 0.26, *SE* = 0.03, 95% CI: 0.23–0.29), but not AS (*p* = 0.199, *b* = 0.02, *SE* = 0.01, 95% CI: −0.01 to 0.04). Widening the confidence interval did not change results.

## 4 Discussion

The current study examined whether AS and DT predicted longitudinal misophonia symptoms. Both AS and DT were generally associated with misophonia symptoms in bivariate analyses. Effects for AS and DT were largely attenuated when adjusting for trait-level misophonia, obsessive-compulsive symptoms, and within- and between-person emotion dysregulation, except AS remained a significant predictor of misophonia symptoms during triggers. The adjusted *p*-value using a BH correction was non-significant for AS. Exploratory parallel mediation models suggested that effect attenuation for DT is likely explained by emotion dysregulation as a mediator of the relation between DT and misophonia, while AS is better conceptualized as a direct effect given that emotion dysregulation did not mediate the relation between AS and misophonia.

Relations between AS and DT were within expected directions when considering these variables in isolation, consistent with extant literature demonstrating links between these transdiagnostic outcomes and misophonia (Schadegg et al., 2021). Attenuation of relations between AS/DT and misophonia when these models were considered cohesively likely reflects shared variance. AS was a significant predictor of misophonia symptoms during triggers even controlling for these clinical variables (Aim 3), suggesting that individuals with higher levels of AS may be more likely to experience more elevated misophonia symptoms in response to momentary triggers. This interpretation is further supported by direct effects of AS on misophonia when exploring emotion dysregulation as a non-significant mediator. However, the BH correction facilitated a non-significant *p*-value, suggesting replications are needed, additional psychological outcomes (baseline misophonia; emotion regulation) are critical to consider, and estimates may vary depending on controls. Distress intolerance may represent a precipitant of heightened emotion dysregulation, in turn contributing to elevated misophonia symptoms.

Individuals with high AS are prone to misinterpreting innocuous bodily sensations as dangerous (e.g., “heart racing is a sign of a heart attack”), theoretically amplifying physiological and emotional arousal (Reiss, 1991). Individuals with misophonia commonly experience physiological arousal when anticipating triggers, such as increased heart rate (Edelstein et al., 2013; Rosenthal et al., 2022; Rouw and Erfanian, 2018). Sensitivity to and the propensity to misinterpret these sensations may exacerbate arousal and worsen misophonia symptoms in the moment.

Although AS is focused on sensitivity to anxiety-related sensations, it is also important to note that anxiety and *anger* share similar markers of physiological arousal, including heightened physiological arousal and have been distinguished by the interpersonal context (i.e., submissive vs. dominance; Russell and Mehrabian, 1974). Past studies have emphasized the dominant role of anger in misophonia (Jager et al., 2020; Rouw and Erfanian, 2018; Shan et al., 2026), with anxiety also commonly identified as a primary or secondary reaction to triggers (Jager et al., 2020; Rouw and Erfanian, 2018). Given the similar physiological arousal patterns observed in anxiety and anger, and potential for these emotions to simultaneously arise in response to triggers, AS may be capturing the sensitivity and catastrophization of emotional arousal more generally. The current findings extend extant research showing AS is associated with anger and aggression in misophonia (Schadegg et al., 2021). AS has also been hypothesized to increase misophonia symptoms through behavioral avoidance strategies, negatively reinforcing avoidance behaviors and potentially amplifying misophonia symptoms (Cusack et al., 2018). It is also possible, however, that AS elicits a primary fear/anxiety-based response, which in turn contributes to a secondary emotional response (i.e., anger), as reductions in AS mediate anger decreases (Thompson and Schmidt, 2021). Additional transdiagnostic factors (i.e., emotion regulation) also appear to play a prominent role.

Clinically, results suggest AS and emotion regulation are potentially promising and clinically malleable targets needing investigation in experimental and treatment-outcome research. AS-based interventions, for example, can be delivered in as little as a single session, with promising effects (e.g., Vujanovic et al., 2012). Relevant to misophonia (e.g., Schadegg et al., 2021; Shan et al., 2026), research has also shown that reductions in AS can improve anger-related outcomes. Thompson and Schmidt (2021) conducted a randomized control trial examining a brief AS reduction intervention (vs. control) in a clinical sample; decreases in AS temporally mediated reductions in anger, hostility and aggression. For individuals with misophonia and high levels of distress intolerance, trials testing enhancing emotion regulation abilities and strategies may be more appropriate.

### 4.1 Limitations and future research directions

The original version of the ASI was used. Results do not allow for a nuanced examination of relations between cognitive, social, and physical dimensions, which would be a next step. The adapted DVMSQ has limited psychometrics beyond multilevel composite reliability estimates here. While the internal consistency alpha for the A-MISO-S was considered acceptable, threshold suboptimal internal consistency may facilitate biased estimates for baseline misophonia severity. Multilevel composite reliability was also suboptimal for the mDERS at the within level.

Although we included a control for obsessive-compulsive symptoms, we did not adjust for general negative affect. Future studies would benefit from incorporating a more comprehensive assessment of stress and negative affect. Our sample is also relatively homogeneous. As a specialized support and advocacy platform, soQuiet may also naturally attract individuals more aware of symptoms, more engaged with misophonia-related resources, or more severely affected. The sample may not be representative of the broader population of individuals experiencing misophonia, including those who experience clinically meaningful symptoms but do not seek support. While the high volume of repeated EMA observations provides precision for within-person estimates, the number of independent participants is modest.

Future research should examine associations between cognitive, behavioral, and physiological responses at all stages throughout misophonia triggers. One potential analytic strategy to incorporate is dynamic structural equation models or autoregressive models accounting for temporality (i.e., affective reactions as a precipitant and outcome from longitudinal misophonia trigger responses). We were also unable to parse event- and interval-contingent triggers; future experience sampling approaches may benefit from collating these data in addition to examining predictors of adherence across completion levels (e.g., high vs. low).

Regarding relations between AS, DT, and misophonia, AS predicted misophonia symptoms in a standalone model, though was no longer significant with a BH correction. Given the relation between AS and DT, the significant association between AS and misophonia may reflect shared variance between both DT and AS, which could explain the null findings in the comprehensive model. Exploratory mediation analyses partially address this concern; replications in larger samples are needed testing transdiagnostic mechanisms simultaneously.

### 4.2 Conclusion

Results provide insight into relations of AS, DT, and clinical symptoms in predicting misophonia symptoms. AS and DT effects were attenuated when adjusting for baseline misophonia severity, obsessive-compulsive symptoms, and momentary emotion dysregulation. The one exception was AS during triggers, wherein higher AS was associated with elevated misophonia (though AS was no longer significant with a BH adjustment). Null DT findings are likely to be attributed to emotion dysregulation as a mediator of the relation between DT and misophonia symptoms. Future research may consider further diversifying samples, using longitudinal assessment, accounting for a broader range of baseline clinical outcome variables, and testing the effectiveness of AS-focused and emotion dysregulation interventions.

## Data Availability

The raw data supporting the conclusions of this article will be made available by the authors, upon reasonable request.
